# LINC01198 promotes proliferation and temozolomide resistance in a NEDD4-1-dependent manner, repressing PTEN expression in glioma

**DOI:** 10.18632/aging.102162

**Published:** 2019-08-30

**Authors:** Wei-Lin Chen, Hong-Jin Chen, Guo-Qiang Hou, Xiao-Hua Zhang, Jian-Wei Ge

**Affiliations:** 1 Department of Neurosurgery, RenJi Hospital, Shanghai JiaoTong University School of Medicine, Shanghai 200127, China

**Keywords:** lncRNA, glioma, PTEN, NEDD4-1, temozolomide resistance

## Abstract

Background: Dysregulation of numerous lncRNAs has been recently confirmed in glioma; however, the majority of their roles and mechanisms involved in this notorious disease remain largely unclear. This study aims to explore the roles and molecular mechanisms of LINC01198 implicated in the proliferation and chemoresistance in glioma.

Results: LINC01198 was elevated in glioma, and this predicted a poorer prognosis for patients with glioma. LINC01198 knockdown inhibited, while LINC01198 overexpression promoted, glioma cell proliferation and resistance to temozolomide. Mechanistically, NEDD4-1 (neural precursor cell expressed, developmentally downregulated 4, E3 ubiquitin protein ligase) and phosphatase and tensin homolog (PTEN) were recruited by LINC01198, which functioned as a scaffold. Moreover, we showed that LINC01198 exerted its oncogenic activities by enhancing the NEDD4-1-dependent repression of PTEN.

Conclusions: Our study elucidated the role of oncogenic LINC01198 in glioma proliferation and temozolomide resistance, and this role may serve as a promising target for glioma therapy.

Methods: LINC01198 expression in glioma tissues and that in paired normal tissues were measured by qRT-PCR. The functional roles of LINC01198 in glioma were demonstrated by a series of in vitro experiments. CCK-8 assay, RNA pulldown, RNA immunoprecipitation and western blotting were used to demonstrate the potential mechanisms of LINC01198.

## INTRODUCTION

Glioma is one of the most aggressive and common primary malignant brain tumors, and it accounts for approximately 80% of all primary malignant brain tumor cases [1–3]. Even though current advances in therapeutic methods for glioma, the median overall survival of the patients is less than 12-18 months after diagnosis due to there being limited therapeutic options [4–6]. Therefore, there is an urgent need to explore the exact molecular mechanisms of glioma progression and developing new and effective treatment strategies to improve patient prognosis.

Long noncoding RNAs (lncRNAs) are a class of RNAs longer than 200 nucleotides in length and without protein-coding capacity. Recently, accumulating studies demonstrate that lncRNAs may be emerging as critical oncogenes in cancer progression [7]. Long intergenic noncoding RNA 1198 (LINC01198), a lncRNA with a gene that is located in chromosome region 13q14.13, was first identified in glioma, and it shows significantly increased expression in tumor tissues; however, its functions and involvement in glioma cells are still unknown [8].

In this study, we assessed LINC01198 expression in glioma and performed functional studies to explore the effects of LINC01198 on glioma progression. Our results demonstrated that LINC01198 expression was upregulated in glioma tissues. The high level of LINC01198 was associated with disease progression and was a predictor for poor prognosis in glioma patients. LINC01198 knockdown turnover resulted in LINC01198-induced glioma cell proliferation and resistance to temozolomide. Moreover, we showed that LINC01198 exerts its oncogenic activities by enhancing NEDD4-1 (neural precursor cell expressed, developmentally downregulated 4, E3 ubiquitin protein ligase)-dependent repression of PTEN (phosphatase and tensin homolog) expression. Taken together, our findings suggest a role of LINC01198 in promoting glioma progression and provide a potential therapeutic target for glioma.

## RESULTS

### LINC01198 is upregulated in human glioma and is correlated with poor prognosis

**Figure 1:**
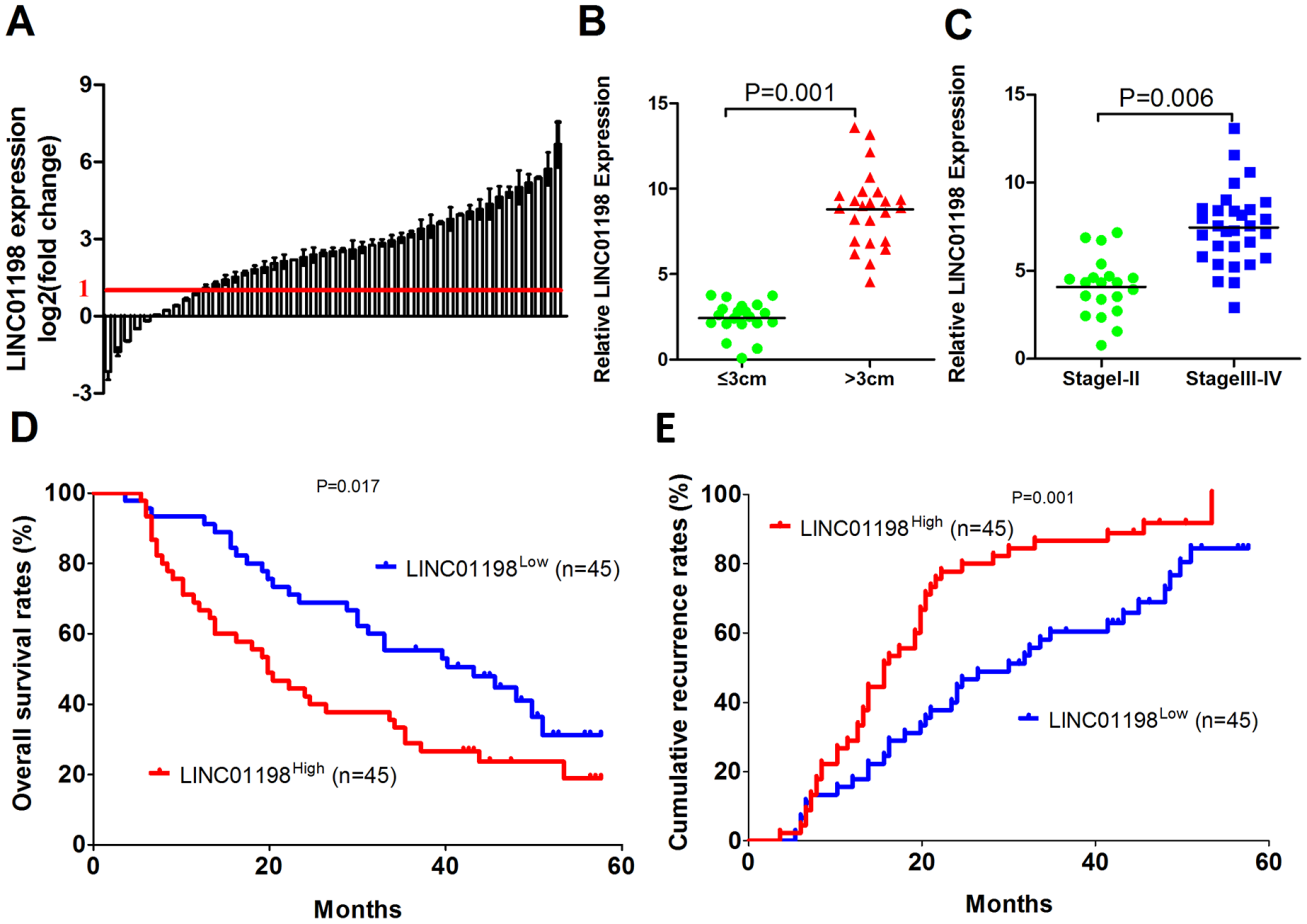
**LINC01198 is overexpressed in human glioma.** (**A**) Expression level of LINC01198 was determined and normalized to GAPDH in 90 pairs of glioma tissues and their corresponding adjacent normal tissues by qRT-PCR. (**B**) The patients were divided into the tumor size ≤3 cm and tumor size >3 cm groups. The diagram shows the LINC01198 expression in each group. P = 0.001. (**C**) The patients were divided into the stage I-II and III-IV groups. The diagram shows the LINC01198 expression in each group. P = 0.006. (**D** and **E**) Glioma patients were divided into the LINC01198^High^ group and LINC01198^Low^ group according to the result of qRT-PCR. Survival and recurrence curves were constructed using the log-rank test. The results show the OS and recurrence for glioma patients with high LINC01198 expression and those with low LINC01198 expression.

Microarray data from GSE16011, Chinese Glioma Genome Atlas (CGGA) and the Repository for Molecular Brain Neoplasia Data (REMBRANDT) datasets show LINC01198 expression increased with tumor grade in glioma [8]. To examine whether LINC01198 plays an important role in glioma progression, we first analyzed the expression level of LINC01198 in human glioma and adjacent normal tissues using qRT-PCR, and we found that the LINC01198 expression level was significantly increased in glioma tissues compared with adjacent normal tissues (74/90) (Figure 1A). Next, we explored the relationship between clinicopathological characteristics and the LINC01198 expression in 90 glioma patients, as listed in Table 1. The results showed that glioma patients with LINC01198^High^ had a bigger tumor size (P = 0.001) and higher WHO grade (P = 0.006) (Figure 1B). Then, we investigated the prognostic implication of LINC01198 expression. Interestingly, our results showed that patients with LINC01198^High^ expression had a worse prognosis than those with LINC01198^Low^ expression (Figure 1C and 1D). Multivariate analysis identified LINC01198 expression as an independent predictor for prognosis of glioma patients (Tables 2 and 3). These results oracle that LINC01198 is likely participated in the progression of glioma.

**Table 1:** Correlation of LINC01198 expression with clinicopathological features in 90 glioma patients.

**Variable**	**LINC01198^High^**	**LINC01198^Low^**	**P**
	**N = 45**	**N = 45**	
Gender			
Female	21	17	0.522
Male	24	28	
Age			
≤ 50	31	29	0.823
> 50	14	16	
Tumor size			
≤ 3cm	14	30	0.001
> 3cm	31	15	
WHO grade			
I–II	17	31	0.006
III–V	28	14	
PRTE*			
≤ 1cm	13	22	0.634
> 1cm	32	23	

*PTBE: Peritumoral Brain Edema.

**Table 2:** Univariate and multivariate analyses of factors associated with overall survival.

**Factors**	**OS**			
	**Univariate, P**	**Multivariate**		
		**HR**	**95% CI**	**P value**
Gender (female vs. male)	0.352			NA
Age (years) (≤50 vs. >50)	0.746			NA
Tumor size (≤3cm vs. >3cm)	0.412			NA
WHO grade (I–II vs. III–IV)	0.007	2.513	0.974–3.152	0.014
PRTE (≤1cm vs. >1cm)	0.441			NA
LINC01198 expression (high vs. low)	0.003	0.821	0.932–1.942	0.008

OS, overall survival; NA, not adopted; NS, not significant; 95%CI, 95% confidence interval; HR, hazard ratio.

**Table 3:** Univariate and multivariate analyses of factors associated with cumulative recurrence.

**Factors**	**OS**			
	**Univariate, P**	**Multivariate**		
		**HR**	**95% CI**	**P value**
Gender (female vs. male)	0.067			NA
Age (years) (≤50 vs. >50)	0.771			NA
Tumor size (≤3cm vs. >3cm)	0.216			NA
WHO grade (I–II vs. III–IV)	0.019	1.982	1.132–3.193	NS
PRTE (≤1cm vs. >1cm)	0.512			NA
LINC01198 expression (high vs. low)	0.004	1.723	0.976–3.004	0.027

OS, overall survival; NA, not adopted; NS, not significant; 95%CI, 95% confidence interval; HR, hazard ratio.

### LINC01198 enhances the proliferation and temozolomide resistance of glioma cells

**Figure 2:**
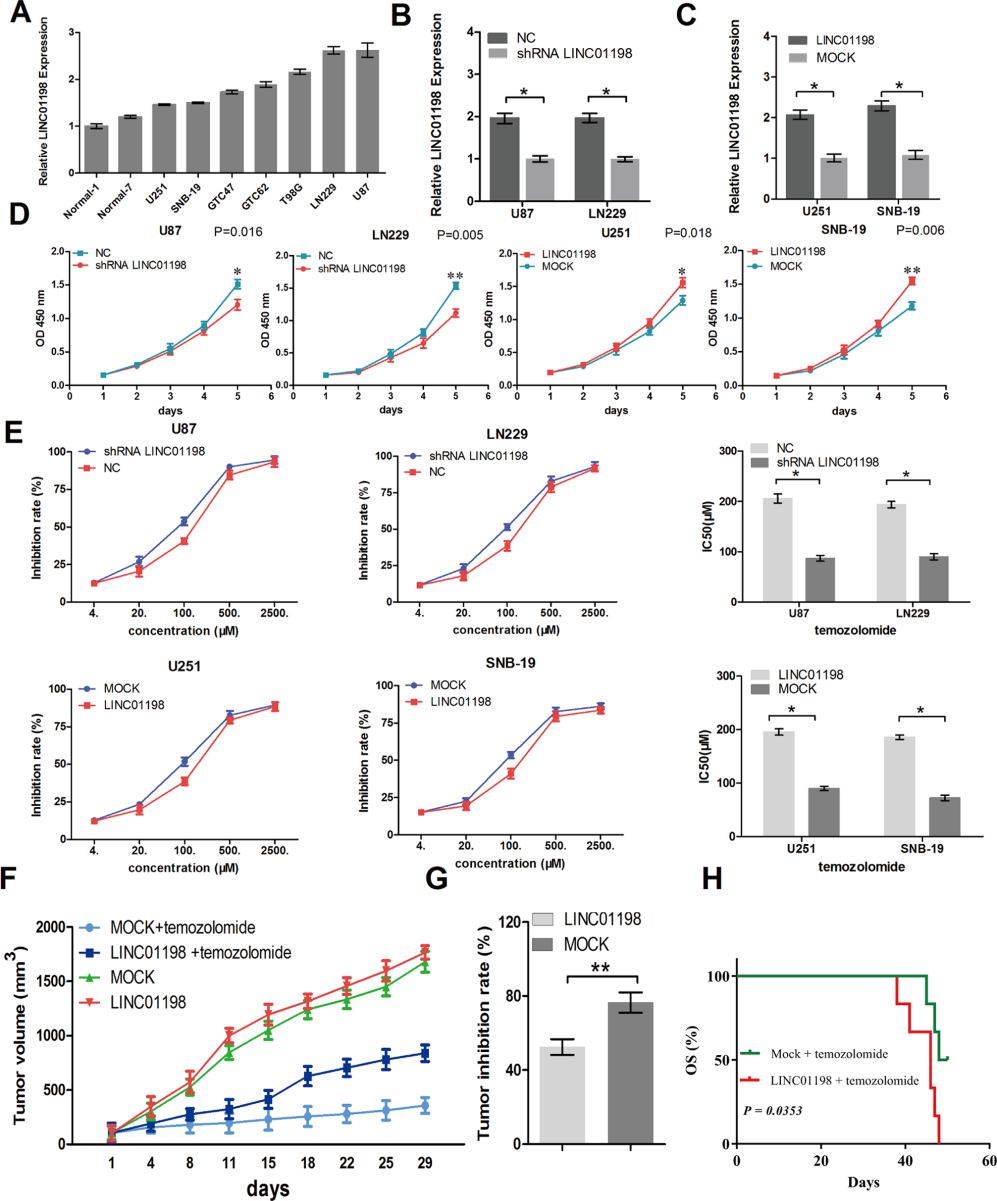
**LINC01198 promotes glioma proliferation and resistance to temozolomide.** (**A**) LINC01198 expression was examined in several glioma cell lines, normal brain tissues, and glioma tissues using qRT-PCR. GAPDH was used as a control for loading. (**B** and **C**) LINC01198 expression in glioma cells was modified by shRNA interference and cDNA transfection. (**D**) Growth curve showing the proliferation activities of glioma cell transfectants in vitro. (**E**) IC50 value of temozolomide on glioma cells. Cells were treated with different concentrations of temozolomide for 72 hours. (**F**) Antitumor effect of temozolomide on glioma xenografts in an established model. (**G**) The data were expressed as percentage inhibition of tumor growth. (**H**) The survival time of mice bearing glioma subcutaneous xenograft received temozolomide therapy. Data are presented as the mean ± SD, *n*=3. *P < 0.05, **P < 0.01.

The biologic function of LINC01198 in regulating the glioma progression remains unclear. Next, we carried out loss/gain-of-function studies in glioma cells. After detecting the expression levels of LINC01198 in six glioma cell lines, two normal brain tissues, and two glioma tissues, we performed knockdown of LINC01198 in U87 and LN229 cells that had high LINC01198 expression and upregulated its expression in U251 and SNB-19 cells that had low LINC01198 expression (Figure 2A–2C). CCK-8 assay result showed that knockdown of LINC01198 impaired glioma cell proliferation, whereas LINC01198 overexpression promoted cell proliferation *in vitro* (Figure 2D). Furthermore, CCK-8 assay was performed to investigate the role of LINC01198 in the sensitivity of glioma cells to temozolomide. The results showed that knockdown of LINC01198 dramatically increased the sensitivity of glioma cells to temozolomide, while LINC01198 overexpression reduced the sensitivity of glioma cells to temozolomide (Figure 2E).

To further investigate the potential clinical relevance of LINC01198 in vivo, we subcutaneously injected U251 cells with or without stable LINC01198 overexpression into the dorsal flanks of 4-6 week BALB/c nude mice. Compared to Mock groups, temozolomide slightly inhibit tumor growth which derived from U251 cells overexpressing LINC01198 xenografts (Figure 2F and 2G). Moreover, the mice bearing subcutaneous xenograft U251 cells overexpressing LINC01198 tumors showed a short survival time compared with Mock groups tumors (Figure 2H).

### LINC01198 functions as a scaffold for NEDD4-1 and PTEN

**Figure 3:**
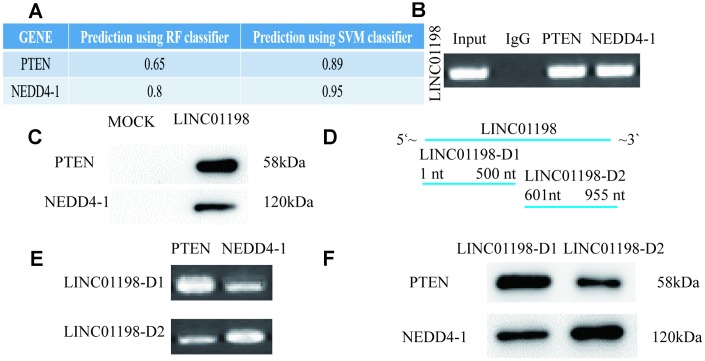
**LINC01198 functions as a scaffold for NEDD4-1/PTEN to regulate PTEN expression in glioma cells.** (**A**) Predicted interaction probabilities of LINC01198 and RNA-binding proteins via a RNA–protein interaction prediction tool. (**B**) RIP assays of LINC01198 binding to indicated proteins in glioma U87 cell extracts. (**C**) RNA pulldown was used to examine the association of LINC01198 and NEDD4-1/PTEN. (**D**) The LINC01198 gene was cut into LINC01198-D1(1-500 nt) and -D2(501-955 nt), and their overexpression vectors were constructed and transfected into U87 cells. (**E**) RIP assays of LINC01198-D1/D2 binding to indicated proteins in glioma U87 cell extracts. (**F**) Biotinylated LINC01198-D1/D2 RNAs were incubated with U873 cell lysates, and western blotting analysis was performed to evaluate the specific association between them and NEDD4-1/PTEN.

To investigate the potential mechanisms of LINC01198 in glioma cells, we predicted the interactions between LINC01198 and RNA-binding proteins by an RNA-protein interaction prediction tool (http://pridb.gdcb.iastate.edu/RPISeq/), and we found that LINC01198 probably binds to NEDD4-1 and PTEN (Supplementary Table 3) (Figure 3A). Then, we carried out RIP assays and found that LINC01198 directly binds NEDD4-1 and PTEN in glioma cells (Figure 3B). In addition, RNA pulldown assays further confirmed that LINC01198 indeed binds with NEDD4-1 and PTEN in glioma cells (Figure 3C). Our results showed that LINC01198 could bind to both NEDD4-1 and PTEN, indicating that LINC01198 may function as a scaffold for NEDD4-1 and PTEN in glioma cells. To further confirm this hypothesis, the LINC01198 gene was cut into LINC01198-D1 (1-500 nt) and -D2 (601-955 nt), and their expression plasmids were constructed and transfected into U87 cells (Figure 3D). Our results showed that LINC01198-D1 mostly bound with PTEN, whereas LINC01198-D2 bound with NEDD4-1 (Figure 3E and 3F). These data showed that LINC01198 may function as a scaffold and bind with PTEN and NEDD4-1 at its 5′ region and 3′ region in glioma cells, respectively.

### LINC01198 increased NEDD4-1-induced PTEN inhibition

**Figure 4:**
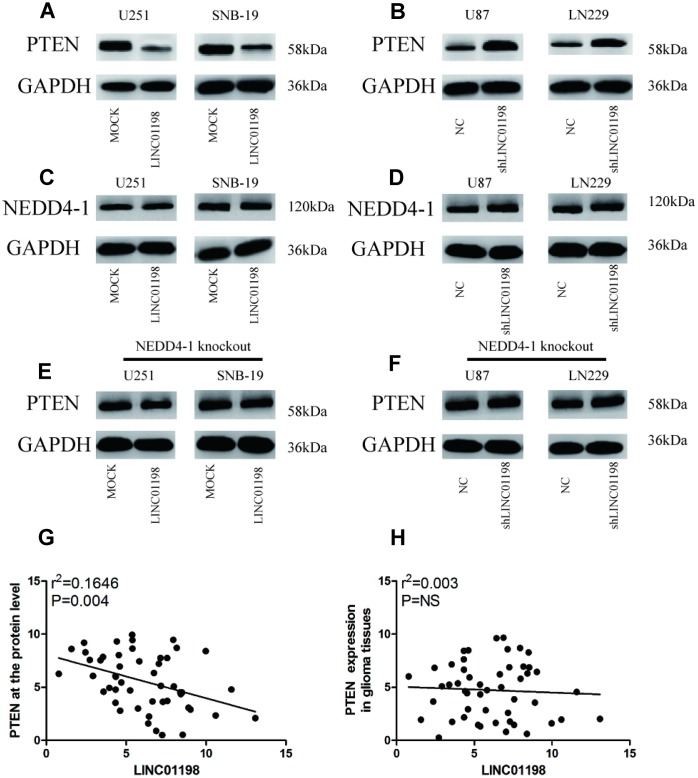
**LINC01198 increased NEDD4-1-induced PTEN inhibition in glioma cells.** (**A**) Forced LINC01198 expression inhibited PTEN expression in glioma cells. (**B**) Protein expression levels of PTEN were increased in glioma cells treated with LINC01198 shRNA. (**C**) Forced LINC01198 expression did not affect NEDD4-1 expression in glioma cells. (**D**) Protein expression levels of NEDD4-1 did not change in glioma cells treated with LINC01198 shRNA. (**E**) Forced LINC01198 expression did not inhibit PTEN expression in CRISPR/cas9-induced NEDD4-1-knockout glioma cells. (**F**) Protein expression levels of PTEN were not increased in CRISPR/cas9-induced NEDD4-1-knockout glioma cells treated with LINC01198 shRNA. (**G** and **H**). A negative correlation between LINC01198 and PTEN was observed in tumor tissues at the protein level (r = 0.501; P = 0.013), but this correlation was not observed at the mRNA level (r = 0.296; P > 0.05).

Previous studies have demonstrated that NEDD4-1-mediated PTEN ubiquitination is crucial in the regulation of PI3K/AKT signaling in neurodegeneration [9]. We thus evaluated the effect of LINC01198 on NEDD4-1-induced PTEN inhibition in glioma cells. Overexpression of LINC01198 significantly inhibited the expression of PTEN protein but not mRNA in U257 and SNB-19 cells (Figure 4A, Supplementary Figure 1A). Conversely, LINC01198 knockdown increased PTEN protein but not mRNA expression in U87 and LN229 cells (Figure 4B, Supplementary Figure 1B). However, in vitro experiments showed that overexpression or knockdown of LINC01198 did not affect NEDD4-1 expression in glioma cells (Figure 4C and 4D). Moreover, overexpression or knockdown of LINC01198 did not affect PTEN expression in CRISPR/cas9-induced NEDD4-1-knockout glioma cells (Figure 4E and 4F; Supplementary Figure 2). Then, to investigate the relationship between LINC01198 and PTEN, we detected the expression of PTEN in 90 cases of glioma tissues using western blotting or qRT-PCR. Scatterplot assays results indicated a negative correlation between LINC01198 and PTEN at the protein level (r = 0.165; P = 0.004; Figure 4G), but this correlation was not shown at the mRNA level (r = 0.003; P > 0.05; Figure 4H). These results indicate that LINC01198 regulates PTEN expression through NEDD4-1.

### NEDD4-1 turnover LINC01198-induced glioma progression

**Figure 5:**
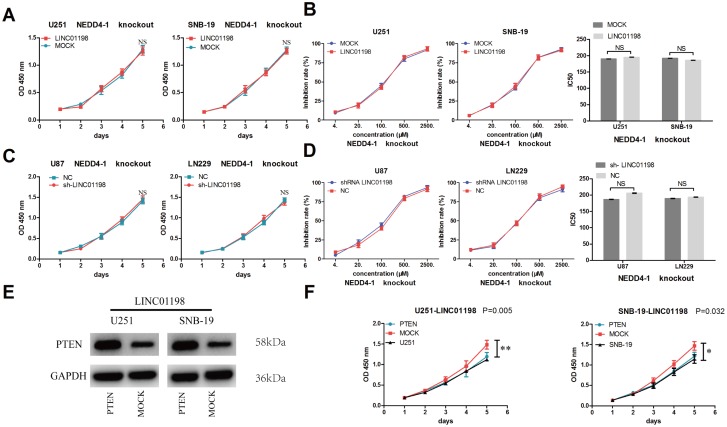
**NEDD4-1 reversed LINC01198-induced glioma proliferation and resistance to temozolomide.** (**A**) Forced LINC01198 expression did not increase cell proliferation in NEDD4-1-knockout glioma cells. (**B**) Forced LINC01198 expression did not promote resistance to temozolomide in NEDD4-1-knockout glioma cells. (**C**) Reduced LINC01198 expression did not inhibit cell proliferation in NEDD4-1-knockout glioma cells. (**D**) Reduced LINC01198 expression did not inhibit resistance to temozolomide in NEDD4-1-knockout glioma cells. (**E**) PTEN expression in LINC01198-overexpressing glioma cells was modified by cDNA transfection. (**F**) recovered PTEN expression reversed LINC01198-induced tumor growth in glioma. Data are presented as the mean±S.D, *n*=3.

To further determine the biological roles of LINC01198 in glioma, CRISPR/cas9-induced NEDD4-1-knockout glioma cells were used in an experiment. A CCK-8 assay showed that forced LINC01198 expression neither increased cell proliferation nor promoted glioma cell resistance to temozolomide in NEDD4-1-knockout glioma cells (Figure 5A and 5B). Additionally, reduced LINC01198 expression neither inhibited cell proliferation nor increased glioma cell resistance to temozolomide in NEDD4-1 knockout glioma cells (Figure 5C and 5D). To further understand the mechanism of LINC01198 in glioma, we recovered PTEN expression in LINC01198-overexpressing cells (Figure 5E). The results showed that recovered PTEN expression reversed LINC01198-induced tumor growth (Figure 5F).

### LINC01198 enhances AKT activity through regulation of the NEDD4-1/PTEN axis

**Figure 6:**
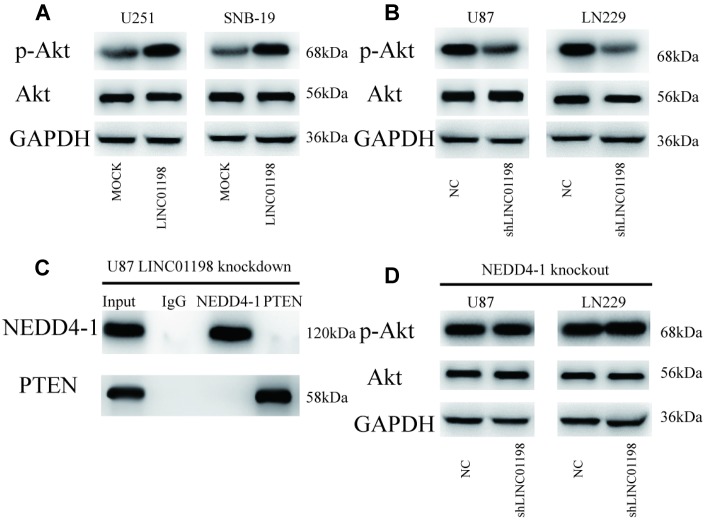
**LINC01198 enhances AKT activity through regulation of the NEDD4-1/PTEN axis in glioma cells.** (**A**) Forced LINC01198 expression upregulated p-AKT expression in glioma cells. (**B**) Protein expression levels of p-AKT were reduced in glioma cells treated with LINC01198 shRNA. (**C**) LINC01198 knockdown inhibited the interplay between NEDD4-1 and PTEN in glioma cells. (**D**) Forced LINC01198 expression did not upregulate p-AKT expression in CRISPR/cas9-induced NEDD4-1-knockout glioma cells.

To explore whether LINC01198 influenced AKT signaling pathway activity, we analyzed the p-AKT expression in glioma cells. As shown in Figure 6A, LINC01198 overexpression increased p-AKT expression. Conversely, knockout of LINC01198 decreased the expression of p-AKT (Figure 6B). In particular, LINC01198 knockdown inhibited the interplay between NEDD4-1 and PTEN (Figure 6C; Supplementary Figure 3). Finally, the LINC01198-induced higher AKT activity was abrogated when knockout of NEDD4-1 was performed (Figure 6D). Collectively, these observations suggest that LINC01198 enhances AKT activity through NEDD4-1-induced PTEN ubiquitin degradation in glioma cells.

### LINC01198 expression is correlated with poor prognosis in glioma patients treated with temozolomide

**Figure 7:**
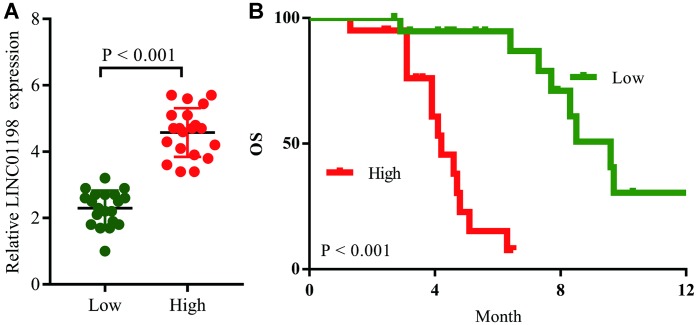
**LINC01198 induces temozolomide resistance in glioma patients.** (**A**) Recurrent glioma patients were divided into the LINC01198^High^ group and LINC01198^Low^ group according to the result of qRT-PCR. (**B**) Comparison of overall survival curves for temozolomide-treated patients with high LINC01198 expression and those with low LINC01198 expression.

**Figure 8:**
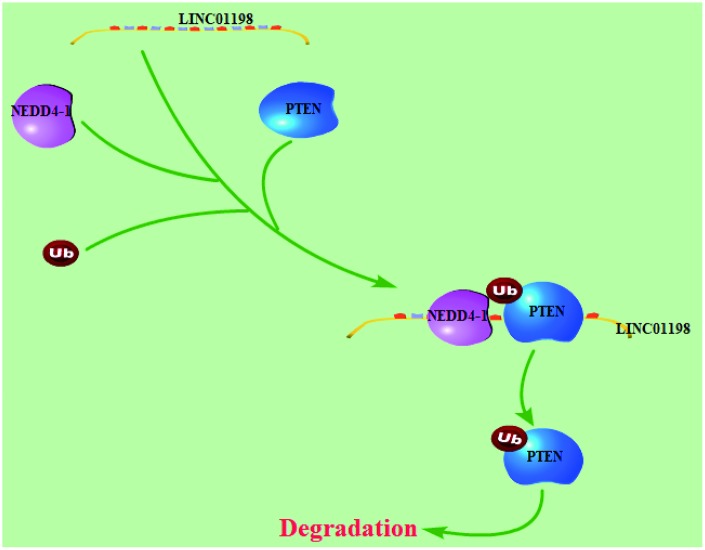
**Working model: LINC01198 overexpression promotes the proliferation and temozolomide resistance of glioma cells.**

We then analyzed retrospective data from 40 recurrent glioma patients after primary tumor resection receiving temozolomide therapy; the clinicopathological characteristics and OS for these patients were recorded in Table 4. Their LINC01198 expression levels were then detected (Figure 7A), and Kaplan–Meier survival analysis showed that the OS for the LINC01198^high^ group was significantly lower than LINC01198^low^ group (Figure 7B). The median OS was 4.7 months in the LINC01198^high^ group, and it was 9.6 months in the LINC01198^high^ group; therefore, we concluded that high levels of LINC01198 led to the temozolomide resistance in glioma patients (Figure 8).

**Table 4:** Correlation of LINC01198 expression with clinicopathological features in 40 recurrent glioma patients.

**Clinicopathologic features**	**Number of patients**
Gender	
Female	18
Male	22
Age	
≤ 50	17
> 50	23
Tumor size	
≤ 3cm	22
> 3cm	18
WHO grade	
I–II	16
III–IV	24
PTBE	
≤ 1cm	15
> 1cm	25

*PTBE: Peritumoral Brain Edema.

## DISCUSSION

Temozolomide is a classic chemotherapeutic drug that is widely used as treatment for glioma. However, the majority of glioma patients exhibit primary or acquired chemotherapy-resistant during temozolomide chemotherapy, which significantly hinders the use of temozolomide. Despite significant advances in the molecular mechanisms of glioma in the last several decades, the exact molecular mechanisms in temozolomide-resistant remain unclearly.

Recently, it has been demonstrated that lncRNAs are dysregulated in many cancers, including in glioma, and they play vital roles in cancer progression through different mechanisms [33, 10–12]. Data derived from the GSE16011, CGGA and REMBRANDT datasets verified LINC01198 overexpression in glioma [8]. However, the biological functions of LINC01198 in glioma have not been reported. In this study, we identified the expression level of LINC01198 and investigated its biological functional role in glioma. Consistent with previous reports, our results demonstrated that LINC01198 expression was upregulated in glioma and that this promoted glioma cell proliferation and chemoresistance to temozolomide. To further explore the molecular mechanism by which LINC01198 plays an oncogenic role, RNA pulldown experiments and RIP were used to determine the downstream target genes. Here, we showed that LINC01198 function as a scaffold to regulate the NEDD4-1-induced ubiquitination degradation process of the PTEN protein at post-transcriptional level.

The tumor suppressor PTEN, an antagonist of the PI3K/AKT pathway, has been found to negatively regulate chemotherapy resistance in several tumors, including glioma [13–15]. An abundance of PTEN is required for the prevention of several cancers and for carcinogenesis [16]. Interestingly, unlike most observed genomic alterations in other cancers, the loss of PTEN in cancer is mainly due to posttranslational modifications [17, 18]. Particularly, E3 ubiquitin ligase-mediated proteasomal degradation of PTEN represents a vital posttranslational mechanism for maintaining a higher level of PTEN in physiological conditions [19]. It has been reported in previous studies that NEDD4-1 and WWP2 are the only two well-defined proto-oncogenic ubiquitin ligases for PTEN degradation; however, PTEN is a stable protein with a long half-life, and only a very robust overexpression of NEDD4-1 can lead to the downregulation of PTEN protein levels in cancer cells [20–22].

PTEN acts as a tumor suppressor gene through inhibition of the PI3K/AKT signaling pathway, which participates in the regulation of various biological functions, including cellular growth, metabolism and survival. Recent studies have confirmed the inactivation of PTEN in several malignancy tumors and leukemia [23–28]. Notch-1 is required for trastuzumab resistance by repressing PTEN expression to contribute to activation of ERK1/2 signaling in breast cancer cells [29]. PTEN status contributes to acquired resistance to BRAF (B-Raf proto-oncogene, serine/threonine kinase) inhibitors in patients who relapse during treatment with BRAF inhibitors [30]. MiR-3142-induced adriamycin resistance occurs through the targeting of PTEN, which leads to downregulation of the PTEN protein and activation of the PI3K/Akt pathway in chronic myeloid leukemia (CML) cells [31]. Here, we showed that PTEN was a RNA-binding protein of LINC01198. Increased of the PTEN protein was detected after inhibition of LINC01198 in glioma cells occurred. Moreover, reduced of PTEN protein expression was detected after up-regulation of LINC01198 in glioma cells occurred. We also found that LINC01198 enhances NEDD4-1-mediated PTEN ubiquitin, leading to the activation of the PI3K/AKT signaling pathway. Our results also indicate that reversing PTEN expression could reverse many of the biological functions of LINC01198. These results indicate that LINC01198 has a critical role in temozolomide resistance by regulating the PTEN/PI3K/AKT signaling pathway.

## CONCLUSIONS

In conclusion, our results indicate that the glioma-associated lncRNA LINC01198 is an oncogenic lncRNA that promotes glioma progression by serving as a scaffold and recruiting NEDD4-1 enzymes to target specific proteins, such as PTEN. Our findings support the idea that lncRNAs, such as LINC01198, play crucial roles in glioma progression, and they suggest that LINC01198 is potentially an effective target for glioma therapy.

## MATERIALS AND METHODS

### Cells and tissue samples

The human glioma cell lines U87, U251, SHG-44, SNB-19, LN229 and T98G were purchased from ATCC (LGC Standards S.r.l.). Cells were cultured in Dulbecco’s Modified Eagle’s Medium (DMEM, Gibco) supplemented with 10% foetal bovine serum (FBS, Gibco) and 1% penicillin/streptomycin (Gibco) in 5% CO_2_ at 37 °C.

Glioma tissues and adjacent tissues were collected from patients with glioma at Renji Hospital from Jan 2008 to Jan 2012. Forty-eight of the tumor samples were pathologically graded as low-grade tumors (stage I and stage II), and 42 were graded as high-grade tumors (stage III and stage IV) according to the WHO criteria. All tissues were preserved in liquid nitrogen. In this study, patient consent was received for all investigations and experiments, which have been approved by the ethics committee for clinical research of Renji Hospital.

### Quantitative real-time polymerase chain reaction (qRT-PCR)

Total RNA was extracted using TRIzol Regentin in accordance with the manufacturer’s instructions (Invitrogen, Carlsbad, CA, USA). Reverse transcription was carried out using the extracted RNA and the M-MLV reverse transcriptase in accordance with the manufacturer’s instructions (Invitrogen, USA). Quantitative real-time polymerase chain reaction (qRT-PCR) analyses were performed using SYBR Premix Ex Taq II (Takara) on an ABI StepOnePlus real-time PCR system in accordance with the manufacturers’ instructions (Applied Biosystems, Foster City, CA, USA). The quantification of the expression of RNA was normalized to the expression of GAPDH. The 2^-∆∆CT^ method was used to evaluate the relative expression. The primers in this study used are listed in Supplementary Table 1.

### RNA immunoprecipitation

The EZMagna RNA immunoprecipitation (RIP) kit (Millipore) was used according to the manufacturer's protocol. U87 cells were lysed in complete RIP lysis buffer, and the cell extract was incubated overnight at 4°C with magnetic beads conjugated with specific antibodies or control IgG. The beads were washed in PBS three times and incubated with proteinase K to remove proteins. Finally, purified RNA was used to perform the qRT-PCR analysis.

### RNA pulldown assay

The RNA pulldown was performed as previously described [32]. RNAs (LINC01198, LINC01198D1, LINC01198D2) were transcribed using T7 RNA polymerase *in vitro*, purified using the RNeasy Plus mini kit, and treated with RNase-free DNase I. Transcribed RNAs were biotin-labeled with the Biotin RNA labeling mix (Ambio Life). Positive, negative, and biotinylated RNAs were mixed and incubated with U87MG cell lysates. Magnetic beads were added to each binding reaction, and this was followed by incubation of the samples at room temperature for 2 hours. Then, the beads were washed with washing buffer, and the eluted proteins were examined by western blotting.

### Western blotting

Western blotting was performed according to standard protocols as described in reference [33]. The antibodies used in this study has added in Supplementary Table 2.

### Proliferation and drug sensitivity analyses

For the proliferation analysis, the effect of the test agents on cell viability was assessed with the CCK-8 assay as described in reference [34]. Three independent experiments were performed. For the drug sensitivity analysis, the cells were seeded into 96-well plates at an initial density of 2 × 10^3^ cells per well. After 24 hours of incubation, the cells were incubated in fresh culture medium containing different concentrations of temozolomide for 72 hours (4μM, 20μM, 100μM, 500μM, 2,500μM). The CCK-8 assay was performed to analyze cell viability.

### Plasmid construction and cell transfection

The full-length complementary cDNA strands of human LINC01198, LINC01198-D1, and LINC01198-D2 were synthesized by Invitrogen and cloned into the expression vector pCDNA3.1. The small hairpin RNA (shRNA) of LINC01198 was provided by Genepharm. Co. Plasmid vectors for transfection were prepared using DNA Midiprep kits and transfected into glioma cells using Lipofectamine 2000 according to the manufacturer's instructions.

### Creation of CRISPR/Cas9-mediated knockout cell lines

Knockout of NEDD4-1 was performed by CRISPR/Cas9 technology, using a previously published protocol as reference [35].

### Subcutaneous xenograft tumor models

Glioma cells (2 × 10^5^) were subcutaneously injected into 4-6 weeks old nude mice. When the tumor volumes reached an average of approximately 100 mm^3^, mice were randomly assigned to treatment groups and vehicle solution group. For treatment groups, temozolomide 25 mg/kg i.p. every 2 days for 4 weeks. The tumor volumes were measured twice every week in two dimensions with Vernier calipers. The tumor volumes were calculated using the following formula: length × width^2^ × 0.5. The mice are considered dead when the tumor volumes reached 2, 000 mm^3^. Mice were housed according to protocols approved by the Medical Experimental Animal Care Commission of Renji hospital.

### Statistical analysis

All analyses were performed using SPSS software (version 16.0). For continuous variables, the results were shown as mean ± SD. Student’s t-test was used to compare the difference between two groups. Differentially expressed LINC01198 between glioma and normal tissues was also evaluated by Student’s t-test. Kaplan-Meier curve and log-rank test were used to evaluate the effect of LINC01198 on survival of glioma patients. A two-sided P value < 0.05 was considered as statistically significant.

## Supplementary Material

Supplementary Figures

Supplementary Tables
