# Supplementary material for: LINC01198 promotes proliferation and temozolomide resistance in a NEDD4-1-dependent manner, repressing PTEN expression in glioma
Source: Aging (Albany NY). 2019 Aug 30;11(16):6053–68. doi: 10.18632/aging.102162 (PMC6738407; doi:10.18632/aging.102162)
Supplement: Supplementary Figures [file aging-11-102162-s002.pdf]

## SUPPLEMENTARY FIGURES

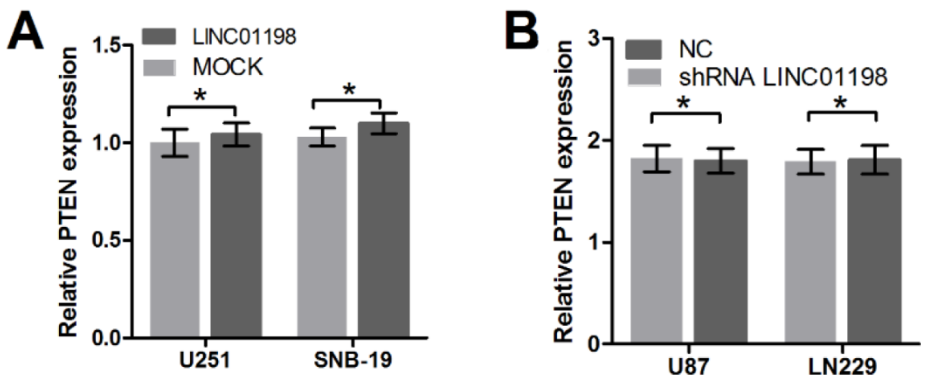


**Supplementary Figure 1. Increased or decreased LINC01198 did not affected PTEN RNA expression in glioma cell lines.**

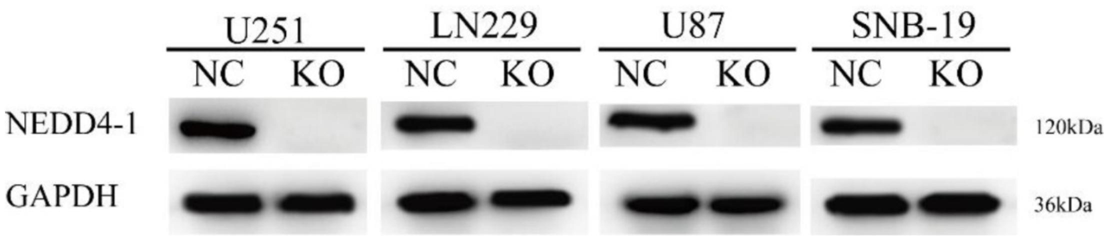


**Supplementary Figure 2. Western blotting was used to verify the CRISPR/Cas9-induced NEDD4-1 knockout efficiency.**

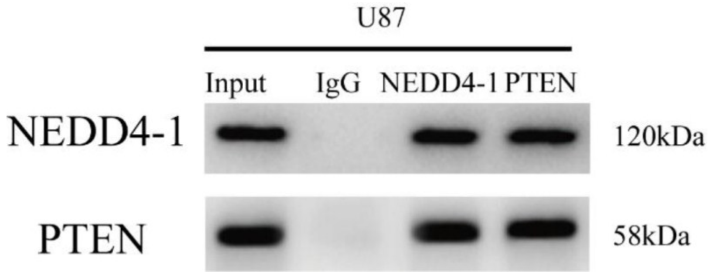


**Supplementary Figure 3. Interplay between NEDD4-1 and PTEN in glioma U87 cells.**
