# Supplementary material for: LINC01198 promotes proliferation and temozolomide resistance in a NEDD4-1-dependent manner, repressing PTEN expression in glioma
Source: Aging (Albany NY). 2019 Aug 30;11(16):6053–68. doi: 10.18632/aging.102162 (PMC6738407; doi:10.18632/aging.102162)
Supplement: Supplementary Tables [file aging-11-102162-s001.pdf]

SUPPLEMENTARY TABLES

Supplementary Table 1. Sequence of primers for qRT-PCR.

Gene	Forward primer (5′-3′)	Reverse primer(5′-3′)
LINC01198	ATGGAGGTGATCACCGTGTAC	GCTGGACGATAACAATGGCAG
GAPDH	GGGGCTCTCCAGAACATCATCC	ACGCCTGCTTCACCACCTCTT

Abbreviations: qRT-PCR, quantitative real-time polymerase chain reaction.

Supplementary Table 2. Antibody for western blotting and RIP used in this study.

Antibody	Company	Cat No.
PTEN	Abcam	ab79156
NEDD4-1	Abcam	ab240753
AKT	Abcam	ab179463
pAKT	Abcam	ab8805
HRP-labeled Goat Anti-Rabbit IgG(H+L)	Beyotime	A0208
HRP-labeled Goat Anti-mouse IgG(H+L)	Beyotime	A0216
GAPDH	Abcam	ab8245

Supplementary Table 3. Predicted the interactions between LINC01198 and RNA-binding proteins.

GENE	Prediction using RF classifier	Prediction using SVM classifier
PTEN	0.65	0.89
NEDD4-1	0.8	0.95
IGF2BP1	0.9	0.68
IGF2BP3	0.9	0.85
BCL2	0.9	0.78
BNDF	0.85	0.81
PTPN1	0.75	0.87
GPIHBP1	0.75	0.8
PAX3	0.9	0.76
HMGA2	0.8	0.65
HMGB1	0.75	0.81
LRIG3	0.85	0.94
PTBP1	0.65	0.87
